# Interactions of Casein and Polypeptides in Multilayer Films Studied by FTIR and Molecular Dynamics

**DOI:** 10.3390/polym11050920

**Published:** 2019-05-25

**Authors:** Lilianna Szyk-Warszyńska, Katarzyna Raszka, Piotr Warszyński

**Affiliations:** Jerzy Haber Institute of Catalysis and Surface Chemistry PAS, ul. Niezapomianjek 8, 30-239 Krakow, Poland; katarzyna.kilan@gmail.com (K.R.); ncwarszy@cyf-kr.edu.pl (P.W.)

**Keywords:** casein, layer-by-layer, protein adsorption, multilayers, polypeptides, FTIR-ATR, molecular dynamics simulations

## Abstract

Multilayer films containing α- and β-casein and polypeptides, poly-L-lysine (PLL), and poly-L-arginine (PLArg) were formed by the layer-by-layer technique and Fourier Transform InfraRed spectroscopy with Attenuated Total Reflection (FTIR-ATR) and FTIR/Grazing Angle analyzed their infrared spectra. We investigated the changes of conformations of casein and polypeptides in the complexes formed during the build-up of the films. To elucidate the differences in the mechanism of complex formation leading to various growths of (PLL/casein)_n_ and (PLArg/casein)_n_ films, we performed the molecular dynamics simulations of the systems consisting of short PLL and PLArg chains and the representative peptide chains—casein fragments, which consists of several aminoacid sequences. The results of the simulation indicated the preferential formation of hydrogen bonds of poly-L-arginine with phosphoserine and glutamic acid residues of caseins. FTIR spectra confirmed those, which revealed greater conformational changes during the formation of casein complex with poly-L-arginine than with poly-L-lysine resulting from stronger interactions, which was also reflected in the bigger growth of (PLArg/casein)_n_ films with the number of deposited layers.

## 1. Introduction

Extensive research effort has been devoted to the study of new materials for biotechnological or biomedical applications in the last few years. Among these materials, the multilayer polyelectrolyte (PE) films have attracted the attention of many scientists [1,2]. Multilayer films can be easily modified by the type of polyelectrolytes used or by species that are embedded in their structure, like nanoparticles, nanocapsules, or proteins. The formation of multilayer films is electrostatically driven, and the free energy gain in that process is due to the release of counterions of polyelectrolyte chains. However, other interactions, such as hydrogen bonding or the hydrophobic effect, can contribute to the formation of films [1].

The adsorption of proteins on a substrate surface usually provokes the conformational changes in their structure that can be either positive, leading to better stability of the whole film, or negative, resulting in the loss of biological activity of the protein. Caseins, which belong to the Intrinsically Unstructured Proteins (IUP), do not adopt stable, folded structures in their natural state [3,4,5]. Consequently, they can change their conformation in an aqueous solution behaving as flexible, disordered, polyelectrolyte-like molecules that can be easily integrated into polyelectrolyte film as one of its components [6,7,8].

Three forms of peptides, α_s_-, β-, and κ-casein, exist in milk casein. Two types of α_s_-casein, α_s1_- and α_s2_-casein, amount to 40% and 10% of the total casein content of milk, respectively, whereas, β - casein comprises 38% [9,10]. Casein has excellent biocompatibility and biodegradability and it has pH-responsive structural, conformational, and functional changes [11,12]. It possesses separated hydrophobic and hydrophilic domains [13]; thus, as a natural polymeric surfactant, it is a good candidate for the preparation of novel drug delivery vehicles [14,15,16]. An open structure, which is a characteristic feature of IUP, originates from the presence of prolines, 17 in α_s1_-casein and 35 in β-casein, being responsible for the preservation of the structure, even after ligand binding [10].

**Figure 1:**
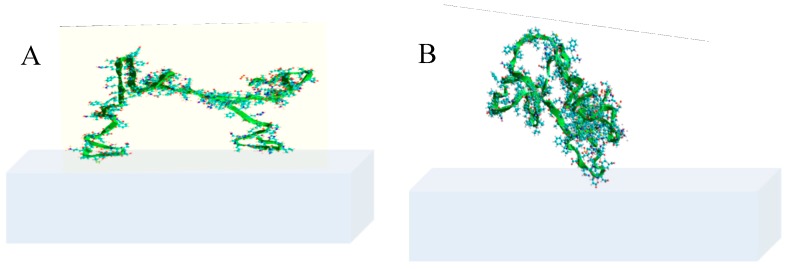
Schematic diagrams depicting typical conformations of (**A**) α-casein and (**B**) β-casein adsorbed onto a planar hydrophobic interface [18].

In the literature, there are reports regarding the monolayer adsorption of β-casein on hydrophobic solid surfaces, sticking to them by the C-terminal, and exposing the N-terminal to the solution [17]. The α-casein, with its different conformation when compared to β-casein, attaches to the surface by the ends of a chain and the middle part of a chain creates a loop (Figure 1) [18].

Casein possesses several advantages, particularly for oral delivery applications, such as high hydrophilicity and biocompatibility [19,20,21]. The absence of toxicity and the presence of reactive sites for chemical modification make it favourable for the development of hydrogel biomaterials [22].

Casein can be applied in biomedical applications to promote biomineralization, as one of its primary functions is the ability to bind calcium ions via phosphate groups of phosphoserine residues or carboxylate groups of acidic amino acid residues [9,23,24]. It can also be used as biosorbent for the removal of toxic heavy metal ions, like zinc, cadmium, mercury, or chromium [25].

In the literature, extensive research has been devoted to the structure investigations of α- and β-casein and the influence of various physicochemical parameters, like pH [26,27], ionic strength [17], temperature [28], and the presence of metallic ions [25,28]. Most of these works described the phenomena that take place in aqueous solutions. Much fewer works have been focused on the adsorption of caseins at solid surfaces [26,29,30,31] and the formation of polyelectrolyte multilayers [8]. In our former work [7], we demonstrated that the ionic strength of solutions of casein and polyelectrolyte influenced the thickness of poly-L-lysine(PLL)/casein films. It was shown that the optimal ionic strength for maximizing the build-up of the (PLL/α-cas)_n_ and (PLL/β-cas)_n_ multilayer films, adsorbed on Si/SiO_2_ supports was *I* = 0.15 M NaCl. A similar observation was described in the work of Svensson et al. [26], where the investigated thickness of the adsorbed chains of β-casein at silica surfaces showed the maximum at 0.1 M of NaCl. That was explained by the stretched conformation of polyelectrolyte and lateral repulsive electrostatic interactions for lower salt concentration, and the screening of the electrostatic interactions between polycation and casein at high salt content. Accordingly, in our following work, we used the same optimal salt concentration (0.15 M NaCl) to form multilayer films consisting of poly-L-arginine (PLArg) and α- or β-casein [32].

**Figure 2:**
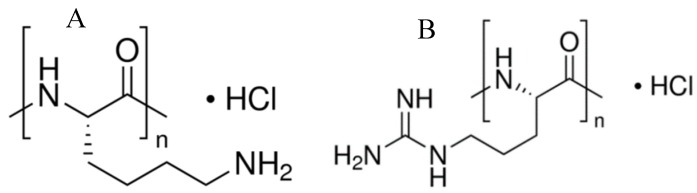
Structures of (**A**) poly-L-lysine (PLL) and (**B**) poly-L-arginine (PLArg).

We selected PLL and PLArg (see Figure 2 for the molecular structures) synthetic polypeptides as cationic biocompatible polymers for multilayer construction. PLL consists of the amino acid lysine, which has an amine group at the end of the side-chain (Figure 2A). It is widely used as a coating to enhance cell attachment and adhesion to solid substrates [33]; it forms complexes with DNA and it has application as a gene delivery tool [34]. PLL zeta potential varies between +27 mV to +19 mV in the range of pH 2 to 9 and it decreases to +9 mV for pH 11 [7]. Poly-L-arginine is a synthetic polyaminoacid having a guanidine group at the end of the side-chain. As a biocompatible polymer soluble in water, PLArg was used as a component of microcapsules for protein or anticancer drug carriers [35]. It can aid as the immunostimulant in the anticancer vaccine [36], and its applications also include the complexation with nucleic acids as sensor components [37] or for gene expression [38]. The zeta potential of PLArg is around +30 mV in the range of pH 2 to 10. Its zeta potential decreases to +8 mV for pH 12 [32].

Poly-L-arginine and poly-L-lysine serve as model cell penetrating peptides (CPP) [39,40,41]. Despite the similarity of ionic properties, interactions with the phospholipid membranes and their penetration are distinctly different [42]. That difference was attributed to the strong electrostatic interactions of arginine with phosphorylated residues [42,43,44]; however, the role of hydrogen bonds [45] and the formation of Arg-phosphate clusters was also considered [46]. As a result, PLArg, as a polypeptide-type gene carrier, exhibits higher transfection efficiency than PLL [47,48].

We focused on the comparison of the interaction of α- and β- casein with the PLL and PLArg adsorbed in the multilayers on the solid surfaces in the present paper. We recorded the FTIR spectra of α and β-casein in KBr pellets and in the deposited casein films and compared them to the spectra of caseins in (poly-L-lysine/casein)_n_ and (poly-L-arginine/casein)_n_ multilayer films that are adsorbed on germanium crystals to elucidate the conformational changes of caseins and polyelectrolytes that are induced by their adsorption at solid surface and at soft support of multilayer films. When comparing the maxima of absorbance in the fingerprint regions, we correlated them with the masses of adsorbed polypeptides and caseins determined by Quartz Crystal Microbalance with Dissipation monitoring (QCM-D) and ellipsometry techniques. We performed molecular dynamics simulations of the interactions of selected casein fragments with short PLArg or PLL chains to explain the observed differences of PLL- and PLArg-casein multilayer films properties and to elucidate the mechanism of observed conformational changes. Since the molecular dynamics simulations of the formation of multilayer films are not possible due to the computational limitation (so are simulations of entire casein—molecules and polyelectrolytes in bulk), we decided to investigate the aggregation of selected casein fragments of 21–25 aminoacids and short PLArg/PLL chains. The nature of interactions between those components in the aggregates and during the formation of polypeptide/casein multilayers are the same. Thus, our simulation results, although preliminary, can shed some light on the origin of the observed differences in PLArg and PLL interactions with phospholipid membranes [42,46,47], whereas the studies of structure changes of proteins adsorbed or embedded in multilayer films can explain the mechanism of protein activity variations. Thus, we consider the application of a combination of molecular dynamics simulations and multilayer films analysis for the characterization of casein-polypeptide interactions as a major novelty of our work.

## 2. Materials and Methods

### 2.1. Materials

The α-casein (Cat. No. C6780-1G, min.70%, α-cas) and β-casein (Cat. No. C6905-1G, min.90%, β-cas) from bovine milk, poly-L-lysine hydrochloride (PLL), MW 30k Da–70 kDa, (Cat. No. P-2636), poly-L-arginine hydrochloride (PLArg) MW 15 kDa -70 kDa, (Cat. No. P7762), and HEPES (N-2-Hydroxyethylpiperazine-N’-2-ethanesulfonic acid, Cat. No. H6147) were obtained from Sigma-Aldrich (Sigma-Aldrich Sp.z.o.o., Poznan, Poland). Sodium chloride pure p.a., hydrochloric acid, sodium hydroxide, were obtained from POCh, Gliwice, Poland. Germanium crystals were purchased from Specac (http://www.specac.com, Orpington, Kent, UK,) as 25 Reflection ATR Germanium crystal (45° angle) as an FTIR-ATR accessory.

Ge crystals before the multilayer film deposition were polished with the 0.05 µm sol-gel alumina suspension (MasterPrep Alumina Suspension from Buehler, Lake Bluff, IL, USA), rinsed with an excess of distilled water, and then dried with a stream of argon.

Polyelectrolyte multilayer films of PLL or PLArg and casein were formed on the supports from the concentration of 0.1 g/dm^3^ of HEPES buffer (c_HEPES_ = 10 mM in 0.15 M of NaCl, pH = 7.4) while using the layer-by-layer technique. The first film layer was always built with PLL or PLArg as a polycation, as all of the supports were negatively charged in pH 7.4. Subsequently, the α- or β-casein was adsorbed as a polyanion. HEPES buffer was used in the washing steps. Further details of the multilayer film formation method can be found elsewhere [7,32].

### 2.2. Method

#### 2.2.1. Fourier Transform Infrared-Attenuated Total Reflection

Fourier Transform Infrared Spectroscopy with Attenuated Total Reflection allows for collecting infrared spectra in an aqueous environment. The infrared spectroscopy is based on the absorption of radiation due to vibrations bonds of molecules. Thus, it provides information regarding their chemical composition and conformational structure [49]. We studied the conformational changes induced by the formation of casein/polyelectrolyte complexes in multilayer films (poly-L-arginine/casein)_n_ and (poly-L-lysine/casein)_n_ adsorbed on a solid support. The region from 1800 to 800 cm^−1^, which is known as the fingerprint region of the infrared (IR) spectrum, is useful for the analysis of protein structure [50], because of the absorption of the bonds forming the amide group (C=O, N-H, and C-N). The two crucial vibration modes of amides are the amide I vibration (measured in the range from 1700 to 1600 cm^−1^), which is primarily caused by the stretching of the C = O bonds, and the amide II vibration (from 1600 to 1500 cm^−1^), which is caused by the deformation of the N-H bonds and stretching of the C-N bonds [28,51]. The exact frequencies at which these bonds absorb depend on the secondary structure of the proteins or peptides [50,51,52,53,54].

The FTIR-ATR experiments were performed by Thermo Scientific Nicolet iS10 (Waltham, MA, USA) spectrometer equipped with Specac 25 Reflection Variable Angle ATR accessory with Liquid Holder P/N GS 11003 flow cell. The cell was equipped with Ge crystal (25 reflections, trapezoid dimensions 52 mm × 20 mm × 2 mm), which, together with Teflon gaskets on its both sides, formed two closed compartments, where adsorption of polyelectrolytes on the germanium surface took place. The spectra were acquired in the range 800–4000 cm^−1^ with the resolution 4cm^−1^ using Omnic software. First, the background spectrum was collected (128 scans of bare crystal mounted in the flow cell), then the cell was filled with HEPES solution and the reference spectrum was taken, and then the solution of polycation (PLArg or PLL) was introduced. Each PE solution was kept in the cell for 15 min to assure adsorption on the crystal surfaces, and the cell was then flushed with HEPES solution with a rate of 0.2 mL/min. Afterward, the casein solution was allowed to flow in, followed by 15 min adsorption step and rinsing with HEPES. The procedure was repeated until multilayer films with four bilayers were obtained. The measurements were conducted after every adsorption and rinsing step for each deposited layer. As all of the polymer/casein solutions were in HEPES, the results that are presented in this paper were obtained by the subtraction of given spectrum and the spectrum for cell only filled with the rinsing solution. Each experiment was repeated twice. Amid I vibration bands were deconvoluted and the second derivative of the spectra was used for the decomposition of spectra into a sum of Gaussian and Lorentzian peaks using PeakFit software (SeaSolve Software, San Jose, CA, USA).

We used the FTIR/Grazing Angle accessory to measure the spectra of the films that were created from the droplets of the PE and casein solution deposited on Ge crystal in some of the experiments. The Monolayer/Grazing Angle Specular Reflectance Accessory (VariGATR) is a dual purpose sampling device that is designed to provide the possibility of in-situ FTIR monolayer investigation of films at an air/liquid interface and grazing incidence angle measurements of thin films on solid reflective surfaces—in this case, germanium.

#### 2.2.2. Molecular Dynamics Simulations

**Figure 3:**
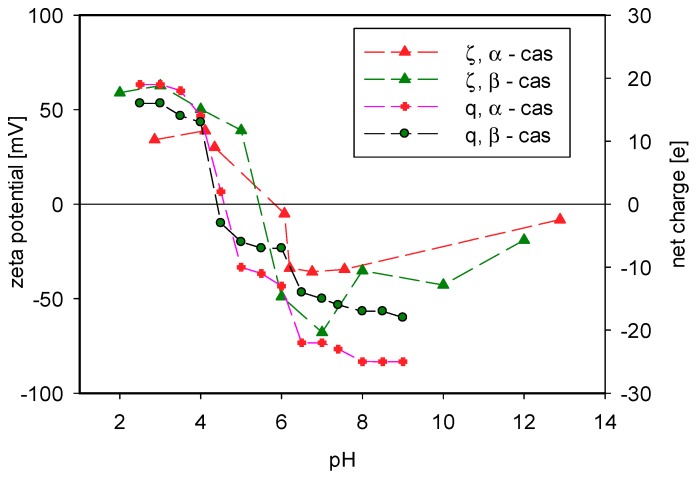
The dependence of zeta potential of α-casein and β-casein on pH value shown together with the calculated net charge of casein molecules obtained with the AutoSMILES procedure [55].

To elucidate the differences in the interactions between caseins and polycations, we performed the molecular dynamics simulations of the system that consisted of short PLL and PLArg chains and polypeptide chains that represent several aminoacid sequences—casein fragments. We used YASARA molecular modeling software [55] with AMBER14 force field [56]. As the starting structures, we used pdb structures of α_s1_-casein [57] and β-casein [58] that were given by Kumosinski et al. The pH-dependent protonation patterns, fractional charges, and bond orders were assigned using the AutoSMILES procedure contained in the YASARA Dynamics package [55]. For the phosphoserine residue, we used the pKa value equal to 5.6 [59]. We calculated the dependence of the net charge of both caseins on pH and compared them with the dependence of their zeta potential measured previously by the microelectrophoretic method to verify the assignment of protonation [32]. Figure 3 illustrates the results.

The calculated dependence of the net charge reflected relatively well the experimental dependence of the casein charge on pH. The value of the isoelectric point for both caseins was only shifted by about one pH unit towards lower values. Moreover, we performed the 100 ns molecular dynamics simulations (conditions are given below) to determine the mean radius of gyration of both caseins. We observed that, after c.a. 30 ns, the radius of gyration attained stationary value with SEM = 2 nm.

Since the formation of the multilayer films is predominantly electrostatically driven, we selected five aminoacid sequences for further processing, three fragments of α_s1_-casein and two fragments of β-casein with various net electric charge. Table 1 lists the selected sequences. Their positions within the casein structure are illustrated in Figure S1 in the Supplementary Materials (SM). The casein fragment was placed at random in the simulation box, 10 nm × 10 nm, together with the PLL or PLArg oligomer consisting of five mers at the beginning of the simulation run. The cell was filled with water molecules (TIP3P, density 1 g/dm^3^) and Na^+^ and Cl^−^ ions to obtain electroneutrality and 0.15 M NaCl salt concentration. The simulation started, the polypeptide-oligomer aggregate was formed, and the energy and the number of hydrogen bonds equilibrated, as illustrated in Figure S2. Subsequently, the simulation ran for 5 ns with the energy and the number of hydrogen bonds sampling every 0.01 ns. No significant differences were observed for longer simulation runs (for 30 s). The electrostatic interactions were calculated using the Particle Mesh Ewald approach [60], while the 1.5 nm cut-off was used for the van der Waals interactions. The procedure was repeated 10 times for every sequence-oligomer combination to account for various binding locations of PLArg/PLL oligomer at the casein fragment. Simulations ran at 295 K. Figure S3 illustrates the example of the changes of the running average of the number of hydrogen bonds during the 30 ns simulation run.

## 3. Results and Discussion

**Figure 4:**
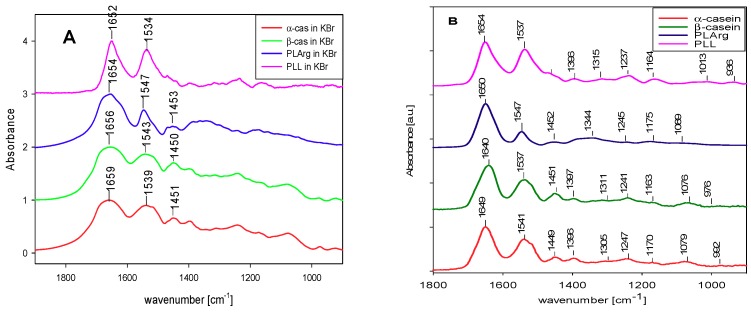
FTIR spectra of α-casein, β-casein, PLL and PLArg (**A**) in KBr and (**B**) dry films on germanium crystal—by VariGATR.

Figure 4A presents the individual IR spectra of α-cas, β-cas, PLL, and PLArg in KBr pellet taken in the transmission mode. For the sake of comparison, Figure 4B shows the FTIR—Grazing Angle spectra of caseins and PE in dry films adsorbed on germanium crystal. Table 2 provides the positions of the maxima of amide vibrations bands. The maxima of the amide bands for polyelectrolytes and casein in films were located in the range between 1640–1654 cm^−1^, red-shifted from the positions of amide I and amide II bands taken in KBr (see Table 2). This shift was more pronounced for caseins (10 cm^−1^ for α-casein and 16 cm^−1^ for β-casein) than for polyelectrolytes, which can indicate on the changes in the secondary structure of casein after adsorption on the solid surfaces.

Decomposition of amide I vibration band spectra, as presented in Figure S4, for both caseins in KBr (part A) and deposited on germanium crystal (part B), revealed an increased contribution of unordered structure conformation of casein chain after the dissolution and formation of a dried film on the solid surface. Assignment of peaks of the spectra to casein secondary structural types, done according to [28,54,61], is provided in Table 3. Following Curley et al. [28], we assigned a peak around 1656 cm^−1^ to large loop structures rather than to α-helices, however, the latter can give some contribution to the spectra at those wavelengths. The results that are presented in Table 3 indicate the increased fraction of random coils and large loops when casein film was formed at Ge crystal.

**Figure 5:**
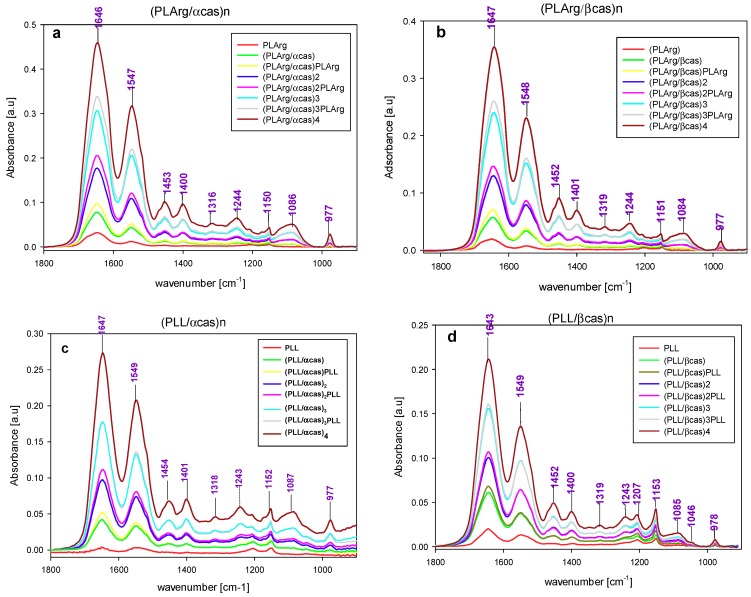
FTIR-ATR spectra of (PLArg/α-cas)_n_, (PLArg/β-cas)_n_—(**a**,**b**) and (PLL/α-cas)_n_ and (PLL/β-cas)_n_—(**c**,**d**) taken after adsorption of the consecutive layer of polyelectrolyte or casein. Numbers denote positions of the maxima for the four bilayers films.

**Figure 6:**
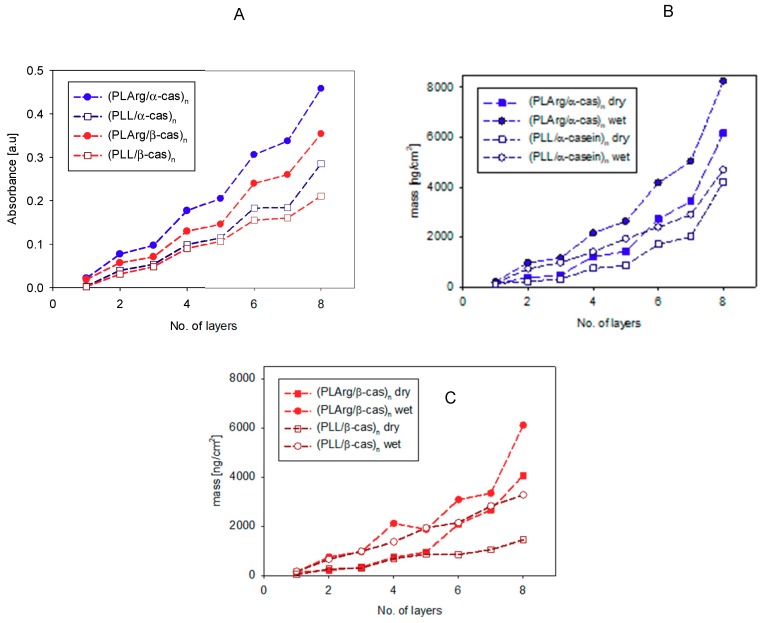
The dependence of the maxima of absorbance value of amide I band vibration of PLArg or PLL and α- or β-casein (**A**), and dry and wet mass of (PLArg/cas)_n_ and (PLL/cas)_n_ films (**B**,**C**) on the number of adsorbed layers (QCM_D and ellipsometry measurements).

**Figure 7:**
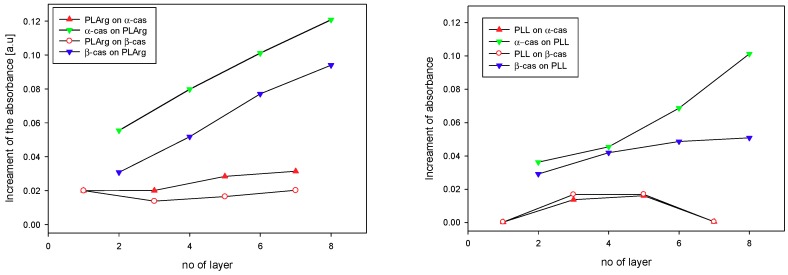
The amid I absorption increments after deposition of the consecutive polypeptide or casein layer during multilayer film formation.

The sequential absorption of multilayers of PLArg with α-cas or β-cas and PLL with α-cas and β-cas, on the silica surface was evidenced by an increase of the absorbance of the amide I vibration band with the number of adsorbed layers, as it was demonstrated in the spectra shown in Figure 5, where grey bars marked the band positions. Figure 6A presents the increase of the maxima of absorbance of amide I band vibration of polypeptide and casein after deposition of the consecutive layers of the film. This increase was more pronounced for PLArg/casein films than for PLL/casein ones. For both polypeptides, larger growth of absorbance with the number of deposited layers was observed for films that were formed with α-casein than with β-casein, which is also illustrated in Figure 7. For PLArg/α-casein film the increment increases from 0.020 to 0.031 for the polymer and from 0.056 to 0.121 for casein, whereas for PLArg/β-casein film from 0.020 to 0.021 for the polymer, and 0.031 to 0.094 for casein, respectively. A similar trend but with a lower increase was observed for films containing PLL. Those results indicated that the films superlinearly grew with the number of deposited layers, as it was demonstrated before by the crystal microbalance with dissipation monitoring (QCM-D) and ellipsometric measurements [7,32]; however, the superlinear increase was caused by growing adsorption of casein at consecutive deposition steps. The comparison of the increase of amid I band intensity with the number of deposited layer with the growth of dry and wet film mass obtained from QCM-D and ellipsometric measurements is illustrated in Figure 6B,C. The same dependence on casein and polyelectrolyte type was observed in all cases.

Larger adsorption was always observed for poly-L-arginine than for poly-L-lysine containing films and both polypeptides produced thicker films (with larger mass) with α-casein than with β-casein. α- and β-casein size can partly explain these differences. Using the molecular structure given in [51,57] the molecular dimension can be directly determined, for α-casein 9 nm and for β-casein 7.5 nm. The molecular dynamics simulations resulted in the average radii of gyration 2.4 nm and 2.05 nm for α-casein and β-casein, respectively. Thus, larger α-casein can create thicker layers comparing with β-casein. Additionally, since, in the QCM-D experiment, the mass of the film is measured together with hydration water; more pronounced growth could be observed for more hydrated α-casein.

Tercinier et al. obtained similar results [31], who observed c.a. 30% larger adsorption of α-casein than β-casein at hydroxyapatite surface. The conformations of α- and β-casein adsorbed on solid and polyelectrolyte surfaces can also bring the explanation to the observed differences. According to the prediction of Horne [18], α-casein can adsorb on the surfaces by the hydrophobic terminals that are situated on both ends of the molecule and the middle part creates a loop (see Figure 1). On the other hand, β-casein adsorbs at positively charged surfaces by the C-terminal and the N-terminal is exposed to the solution. The brushes of N-terminal of β-casein chain are embedded in the PE chain structure and collapse after the adsorption of the subsequent polyelectrolyte layer. The α-casein loops can retain their conformation during the adsorption of the consecutive layers of PE, and in consequence, thicker films can be formed.

As the polypeptides used in our experiments—PLArg and PLL—were of similar molecular weight and they were similarly positively charged at pH 7.4, with the electrostatic interactions being considerably screened at the NaCl concentration 0.15 M, other differences in the complexation mechanism of PLArg and PLL with both caseins during the build-up a multilayer films should be proposed. By the molecular dynamic simulations, Li et al. [46] showed that arginine creates more extensive H-bonds with phosphate groups of lipids than lysine due to higher number of amine groups and the formation of “bi-dentate” hydrogen bonds, i.e., a pair of adjacent and parallel hydrogen bonds that are enabled by molecular geometries of interacting guanidine and phosphate groups. Moreover, taking into the account that α-casein has 8, whereas β-casein possess only five phosphoserine residues, that can explain why PLArg with α-casein create thicker films that with β-casein and why these films are thicker than those build with PLL.

**Figure 8:**
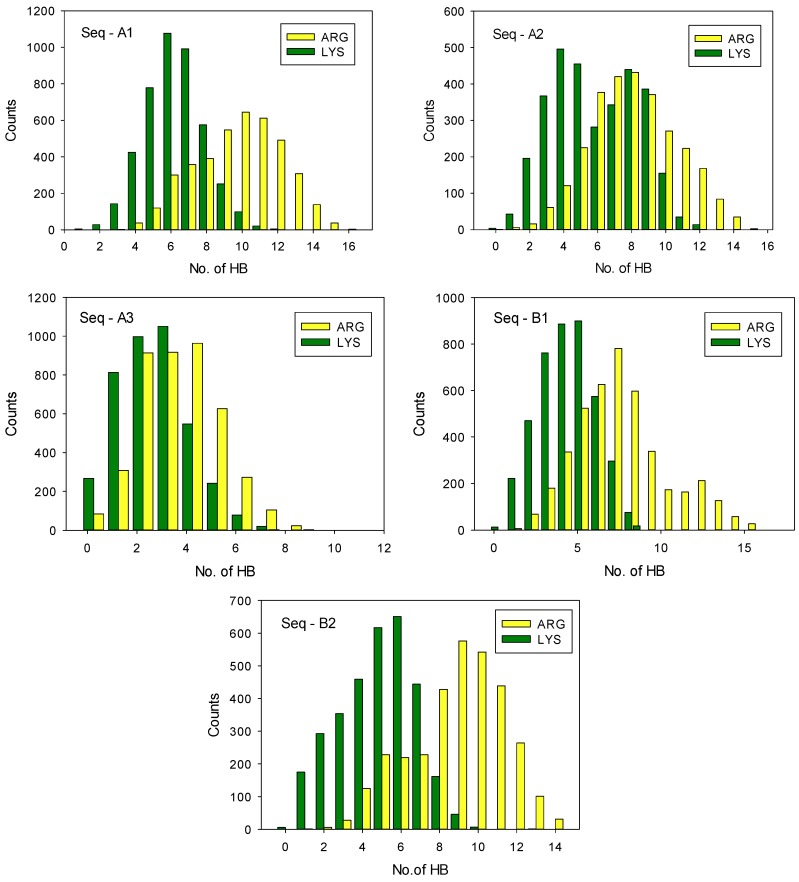
Histograms of the number of hydrogen bonds during molecular dynamic simulation of polypeptide-casein fragment complexes, calculated with 10 replica of the random initial position of casein fragments and PLArg/PLL oligomers. HB sampling time 5 ns, sampling frequency 0.01 ns.

**Figure 9:**
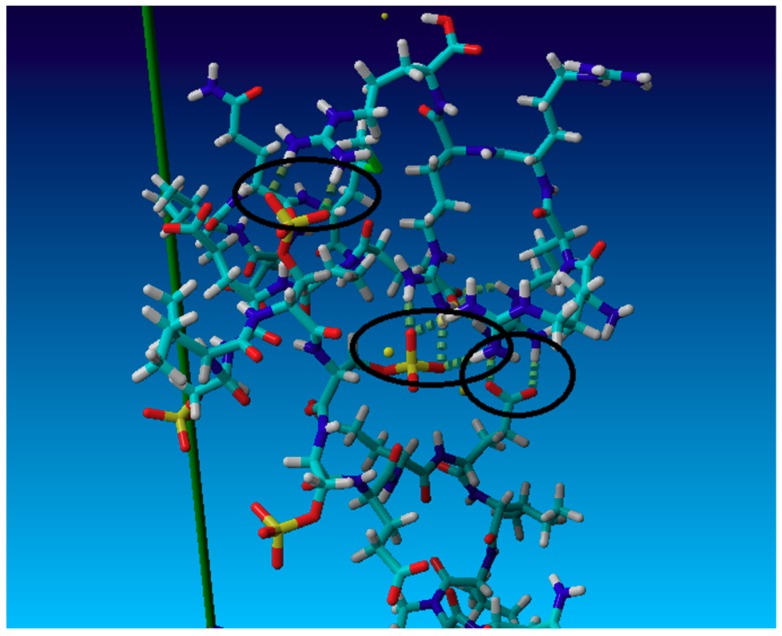
Snapshot from the molecular dynamics simulations illustrating formation of bi-dentate hydrogen bonds between guanidine group of polyarginine and phosphate or carboxyl group of casein.

We performed the molecular dynamics simulations, as described in the previous section, to substantiate that such mechanism can be responsible for the differences in the polypeptide-casein complexation and, consequently, for the formation of multilayer films. They demonstrated that the changes of the electrostatic energy upon the formation of the complex of aminoacid sequences and arginine or lysine oligomers were similar; however, there were significant differences in the number of hydrogen bonding between them. These differences depended on the type of aminoacids in the sequence, as illustrated in Figure 8, where the frequencies of the occurrence of the number of hydrogen bonds during the simulation are shown in the form of histograms. The sampling time used in the simulation was 5 ns and the frequency 0.01 ns. We found that the extension of the sampling time to 30 ns did not make any significant differences in the histograms. They show that there is a significant difference in the number of hydrogen bonds in complexes with arginine and lysine oligomer for all aminoacid sequences. The arginine oligomer formed, on average, one to four hydrogen bonds more than the lysine one, as shown in detail in Table 4. That difference was the most pronounced for the casein segment containing high number of phosphoserine or glutamic acid residues and the difference in the hydrogen bonding energy ranged from 4 to 19 kcal/mol. Those preliminary results of molecular dynamics simulations indicate that poly-L-arginine interacts stronger than poly-L-lysine with both of the caseins due to a stronger tendency to form hydrogen bonds, to a large extent due to the formation of the formation of “bi-dentate” hydrogen bonds with phosphate groups, as illustrated in Figure 9. Moreover, the α_S1_-casein bearing at neutral pH higher negative charge due to 8 phosphoserine, 25 glutamic acid, and 4 aspartic acid residues interacts with both polypeptides stronger to β-casein bearing 5 phosphoserine, 19 glutamic acid, and 4 aspartic acid residues [9]. Most of the works that involved molecular dynamics simulations concerned the differences in the interactions of arginine and lysine residues with phospholipid membranes. Li et al. investigated the interactions of lysine and arginine side chains with model phospholipid membranes. They found that that Arg attracted more phosphate and water in the membrane and formed extensive H-bonding with five H-bond donors to stabilize the Arg-phosphate clusters [46]. Wu et al. used the molecular dynamics (coarse-grain and atomistic) to study the phase behavior of peptide-lipid mixtures. They demonstrated the difference between Arg and Lys that originated from the stronger interactions of arginine with lipid glycerols due to the multivalent nature of the guanidinium group [61]. Here, we show that a similar mechanism determines the interactions between arginine and lysine polypeptides with proteins containing phosphorylated residues.

**Figure 10:**
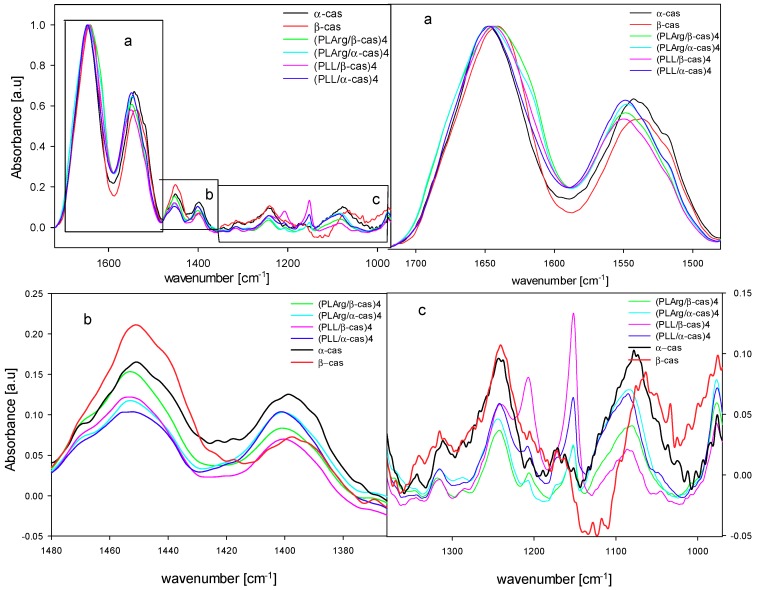
FTIR spectra of free caseins and four bilayer films with PLArg and PLL. Parts a, b, c shows close-ups of part of the spectra illustrated in the top-left figure.

The analysis of the FTIR spectra of films is complex, since both polypeptide and casein contribute to amide vibration bands. The normalized amid I vibration band that is presented in Figure 10 shows the differences in the spectra of studied multilayers films, in particular, at the shoulders of the spectra between 1610–1640 cm^−1^ (Figure 10a). In this region, the β-sheet conformation usually appears, indicating the existence of intermolecular aggregates [62].

We compared the ATR FTIR spectrum of α- and β-casein films directly deposited at the Ge crystal with the differential spectra of casein deposited on top of the multilayer to elucidate the effect of polypeptide-casein interaction on the structural changes of casein. For that purpose, we subtracted the spectra of (casein/PE) bilayer and PE monolayer as well as the spectra of (casein/PE)_4_ and PE(casein/PE)_3_ films. Table 5 and Figures S5 and S6 present the decomposition of amid I vibration bands spectra. The increase of the band with the wavelength around 1615 cm^−1^ was observed that could be attributed to the structural changes that are associated with the formation of aggregates as the result of interactions with underlying polypeptide layer for both caseins at the multilayer film [62]. That increase was more pronounced for the multilayers of caseins with poly-L-arginine.

**Figure 11:**
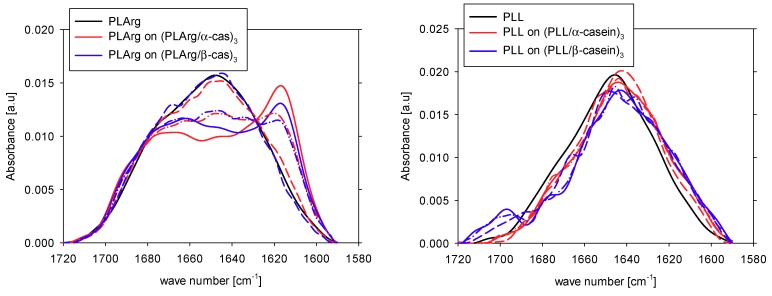
Amid I vibration band for polypeptide film (black) and for polypeptide layer on top of the multilayer film with caseins for poly-L-arginine (red) and poly-L-lysine (blue). The dash, dash-dot and solid lines denote polypeptide on single bilayer, two bilayer, and three bilayer films, respectively.

We also analyzed the FTIR spectra of PLArg and PLL films directly deposited at Ge crystal and compared them with the differential spectra of the same polypeptide layer on the top of the multilayer film. Figure 11 illustrates these spectra and the results of their decomposition for polypeptide films directly deposited at Ge crystal and on top of three bilayer films are collected in Table 6. The remarkable difference in the effect of interaction with caseins was observed. For poly-L-arginine, the amide I band changed its shape and its maximum was shifted to longer wavelengths from 1650 to 1615 cm^−1^. The area of the peak around 1615 cm^−1^ increased from 14% for PLArg film to 34% and 31% of the amide I band for PLArg at α-casein and β-casein multilayer, respectively. This shift was much smaller for PLL, not exceeding 5 cm^−1^, and the area of the peak around 1615 cm^−1^ increased from 8% for PLL film to 18% and 16% at the α-casein and β-casein multilayer. Those results show that the interaction of poly-L-arginine with casein is much stronger than of poly-L-lysine, leading to more pronounced changes in the conformation when polypeptide was adsorbed on casein. Moreover, both of the polypeptides interact stronger with α- then with β-casein. The changes in the spectra gradually occur, depending on the underlying films. For PLArg or PLL deposited on top of single bilayer films, its spectrum is very similar to one at the Ge crystal. That means that, during depositon, polyelectrolytes are mainly covering yet uncoated surface. On the other hand, the spectra for PLArg or PLL deposited on top of two bilayer films are closer to ones for the three bilayer films, thus the interaction of polypeptides with casein determines their structure.

**Figure 12:**
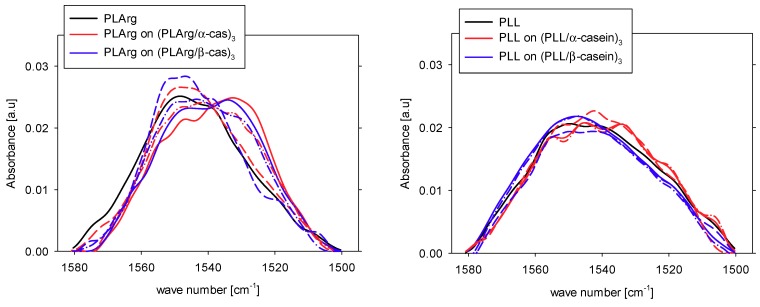
Amid II vibration band for polypeptide film (black) and for polypeptide layer on top of the multilayer film with caseins for poly-L-arginine and poly-L-lysine. Meaning of curves as in Figure 10.

In the amide II vibration band (1600–1490 cm^−1^), a high intensity of absorbance occurred for (PLArg/α-cas)_4_ and (PLL/α-cas)_4_ films, decreased for (PLArg/β-cas)_4_, and attained the lowest value for (PLL/β-cas)_4_ film. That can be due to hydrogen bonding to amide groups [63] and deprotonation of carboxyl groups (COO-) of aspartic and glutamine residues [64,65]. We also compared the spectrum of amide II band for PLArg and PLL films directly deposited at the Ge crystal with the differential spectra of the same polypeptide layer on top of the multilayer film with caseins. The spectra shown in Figure 12 illustrate the same trends as observed for the amide I band. For PLArg that was adsorbed on top of (PLArg/casein) film, the spectrum changes the shape and its maximum is shifted by 16 cm^−1^ for α-casein and by 13 cm^−1^ for β-casein, whereas, for PLL, the shift of spectrum is much smaller.

An interesting observation can be made for the spectra near 1200 cm^−1^, as shown in Figure 10c. There was a big difference in the amplitude of absorption spectra of α- and β-casein (see black and red line in Figure 10c). For PE/caseins films, an additional peak near 1208 cm^−1^ appeared. This observation suggests that phosphate groups, for which part of the spectra for the asymmetric PO_2_^−^ vibrational band are located near 1240 cm^−1^, are involved in the process of polyelectrolyte binding to caseins [66,67]. For PLArg/casein films, the peak amplitude was much lower than for PLL/casein, so this binding is more pronounced in PLArg/casein complexes. The observed shift of phosphate peak located near 1079 cm^−1^ and 1069 cm^−1^ in the spectra of α- and β-casein, respectively, towards higher wavenumbers (by ca. 10 cm^−1^) results from the dehydration of phosphate group and the binding cationic amine group of polyelectrolytes [68,69]. This observation is consistent with the results of molecular dynamics simulation that phosphoserine residues participate in the electrostatic interactions and hydrogen bonding in the formation of casein/polypeptide complexes.

## 4. Conclusions

We showed that ATR FTIR could be conveniently used for studying the conformational changes occurring at the formation of polypeptide, poly-L-arginine and poly-L-lysine, complexes with casein during the deposition of polypeptide/casein multilayer films. Analyzing the absorbance in the amid I vibration band, we demonstrated the superlinear growth of the α-casein/polypeptide or β-casein/polypeptide multilayer films with the number of deposited layers that confirmed the earlier results obtained by the ellipsometry and QCM-D. Adsorption of casein at the surface of germanium crystal induced some conformational changes in casein molecule structure with the preference for random structures and loops. On the other hand, spectra for caseins deposited on the multilayer films indicated on the formation of intermolecular aggregates, more significant for the films built with poly-L-arginine than for ones with poly-L-lysine. The analysis of FTIR spectra of polypeptides adsorbed on casein in the multilayer film evidenced on changes in their conformations in respect to the films deposited directly at Ge surface. Those changes were again much more pronounced for poly-L-arginine than for poly-L-lysine that indicated stronger interaction with caseins. To elucidate the mechanism responsible for the differences in the polypeptide-casein interaction during their complexation and formation of multilayer films we performed the molecular dynamics simulations of the systems consisting of short PLL and PLArg chains and representative polypeptide chains – casein fragments consisting of several aminoacids. They demonstrated that the arginine oligomer formed more hydrogen bonds with phosphoserine and glutamic acid residues than the lysine one, thus, despite similar charge properties at neutral pH, it interacted stronger with casein. Additionally, α-casein bearing at that pH more of phosphoserine and glutamic acid residues interacts with both polypeptides stronger to β-casein. Those preliminary results of molecular dynamics simulation revealed the mechanism of stronger interaction of PLArg with caseins during multilayer film formation explaining their larger growth comparing to PLL containing films. Our results can contribute to the application of casein micelles coated with the cell-penetrating peptides as drug delivery systems.

## Figures and Tables

**Table 1:** Sequences of aminoacids representing fragments of casein chain used in the molecular dynamics simulations.

Casein Type	Sequence Name	Aminoacid Sequence	Aminoacid Number	Net Charge [e]
α_s1_-casein	Seq-A1	ASP-ILE-LYS-GLN-MET-GLU-ALA-GLU-PSE-ILE-PSE-PSE-PSE-GLU-GLU-ILE-VAL-PRO- ASN-PSE-VAL	56–76	−14
	Seq-A2	VAL-ASN-GLU-LEU-SER-LYS-ASP-ILE-GLY-PSE-GLU-PSE-THR-GLU-ASP-GLN- ALA-MET-GLU-ASP-ILE	37–57	−11
	Seq-A3	TYR-THR-ASP-ALA-PRO-SER-PHE-SER- ASP-ILE-PRO-ASN-PRO-ILE-GLY-SER-GLU-ASN-SER-GLU-LYS-THR-THR-MET-PRO-LYS-TRP	173–199	−3
β-casein	Seq-B1	GLY-GLU-ILE-VAL-GLU-PSE-LEU-PSE-PSE- PSE-GLU-GLU-SER-ILE-THR-ARG-ILE-ASN-LYS-LYS-ILE	10–30	−9
	Seq-B2	ILE-ASN-LYS-LYS-ILE-GLU-LYS-PHE-GLN- PSE-GLU-GLU-GLN-GLN-GLN-THR-GLU-ASP-GLU-LEU-GLN-ASP	26–47	−6

**Table 2:** The positions of the maxima of amide absorption bands measured in KBr pellets and in dry film at Ge crystal.

Band Assignment	α-Casein	β-Casein	PLArg	PLL
Amide I in KBr	1659 cm^−1^	1656 cm^−1^	1654 cm^−1^	1652 cm^−1^
Amide I dry film	1649 cm^−1^	1640 cm^−1^	1650 cm^−1^	1654 cm^−1^
Amide II in KBr	1539 cm^−1^	1543 cm^−1^	1547 cm^−1^	1534 cm^−1^
Amide II dry film	1541 cm^−1^	1537 cm^−1^	1547 cm^−1^	1537 cm^−1^

**Table 3:** Decomposition of amide I vibration band spectra of casein, measured in KBr pellets and in dry film at Ge crystal, into structural components.

Band Assignment	α-Casein in KBr		α-Casein Film on Ge		β-Casein in KBr		β-Casein Film on Ge	
	cm^−1^	% area	cm^−1^	% area	cm^−1^	% area	cm^−1^	% area
β-sheet aggregation	1615	7	1615	6	1611	5	1615	9
β-sheet	1630	12	1631	25	1627	15	1633	27
disordered structure	1644	14	1646	24	1644	16	1646	24
large loops	1657	17	1658	20	1657	15	1658	19
turns	1672	22	1671	15	1672	23	1672	14
β-sheet antiparallel	1687	27	1686	10	1692	26	1686	7

**Table 4:** The number of anionic residues, charge, the average number of hydrogen bonds formed by casein fragment with arginine and lysine oligomer and the difference of the hydrogen bonding energy that resulted from the molecular dynamics simulations.

Sequence	No. of PSE	No. of GLU	No. of ASP	Charge [e]	<HB> PLArg	<HB> PLL	ΔE_HB_ [kcal/mol]
Seq–A1	5	4	1	14	10	6	17 ± 4
Seq–A2	2	4	3	10	8	6	12 ± 3
Seq–A3	0	2	2	3	4	3	5 ± 2
Seq–B1	4	4	0	9	8	5	16 ± 4
Seq–B2	1	5	2	6	9	5	18 ± 4

**Table 5:** Decomposition of the amide I vibration band into structural components for casein films and the top casein layers of (PE/casein) and (PE/casein)_4._

**Band assignment**	**Band frequency [cm^−1^]**	**α-cas [%]**	**(PLArg/α-cas) [%]**	**(PArg/α-cas)_4_ [%]**	**(PLL/α-cas) [%]**	**(PLL/α-cas)_4_ [%]**
aggregate intermolecular	1610–1620	6	10	14	9	11
β-sheet	1620–1642	24	19	19	17	21
random coil	1644–1648	24	19	21	23	20
large loops	1654–1658	20	20	23	24	22
β-turns	1667–1685	15	25	15	18	16
β-sheet	1688–1697	10	7	8	9	10
**Band assignment**	**Band frequency [cm^−1^]**	**β cas**	**(PLL/β-cas)**	**(PLArg/β-cas)**	**(PLL/β-cas)**	**(PLArg/β-cas)_4_**
aggregation intermolecular	1610–1620	9	10	15	11	12
β-sheet	1620–1642	27	23	24	23	25
random coil	1644–1648	24	21	20	18	20
large loops	1654–1658	19	23	20	24	21
β-turn	1667–1685	14	17	16	20	18
β-sheet	1688–1697	7	6	5	4	4

**Table 6:** Decomposition of the amide I vibration band into structural components for polypeptide films and the PLArg and PLL layers on top of multilayer films with casein_._

**Band assignment**	**Band frequency [cm^−1^]**	**PLArg [%]**	**PLArg(PLArg/α-cas)_3_ [%]**	**PLArg(PLArg/β-cas)_3_ [%]**
aggregation intermolecular	1610–1620	12	34	31
β-sheet	1620–1642	21	8	10
Arginine	1633			
random coil	1644–1648	19	16	17
large loops	1654–1658	16	8	13
β-turns	1667–1685	21	24	15
Arginine	1673			
β-sheet	1688–1697	10	10	14
**Band assignment**	**Band frequency [cm^−1^]**	**PLL**	**PLL(PLL/α-cas)_3_**	**PLL(PLL/β-cas)_3_**
aggregation intermolecular	1610–1620	8	18	16
β-sheet	1620–1642	22	19	21
Lysine	1629			
random coil	1644–1648	23	30	25
large loops	1654–1658	24	21	20
β-turn	1667–1685	17	10	12
β-sheet	1688–1697	6	2	6

## Supplementary Materials

The following are available online at https://www.mdpi.com/2073-4360/11/5/920/s1.
