# Supplementary material for: Interactions of Casein and Polypeptides in Multilayer Films Studied by FTIR and Molecular Dynamics
Source: Polymers (Basel). 2019 May 25;11(5):920. doi: 10.3390/polym11050920 (PMC6572437; doi:10.3390/polym11050920)
Supplement: Supplementary file 1 [file polymers-11-00920-s001.pdf]

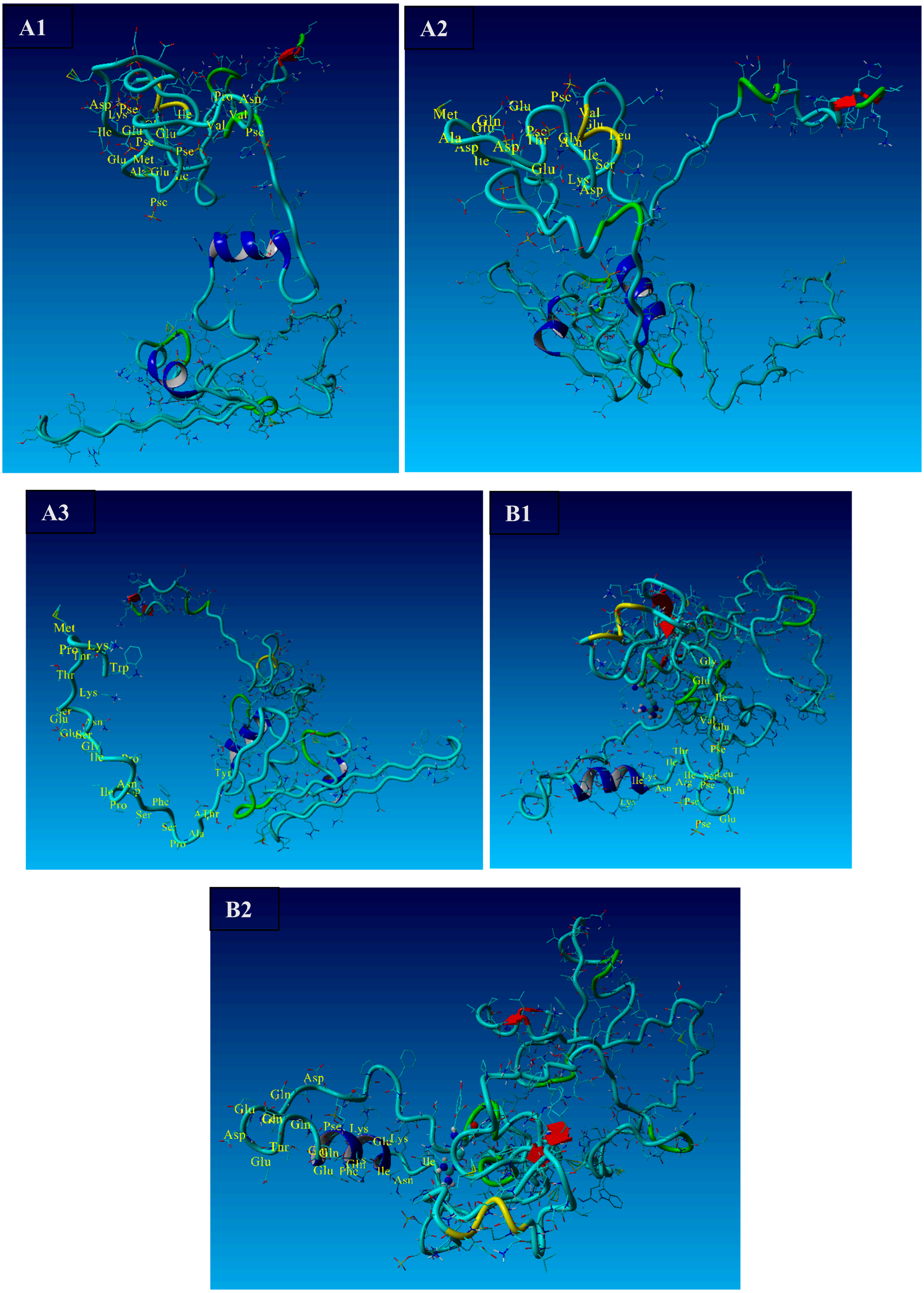

Fig. S1. Illustration of the position of selected aminoacid sequences within α- and β-casein molecule.

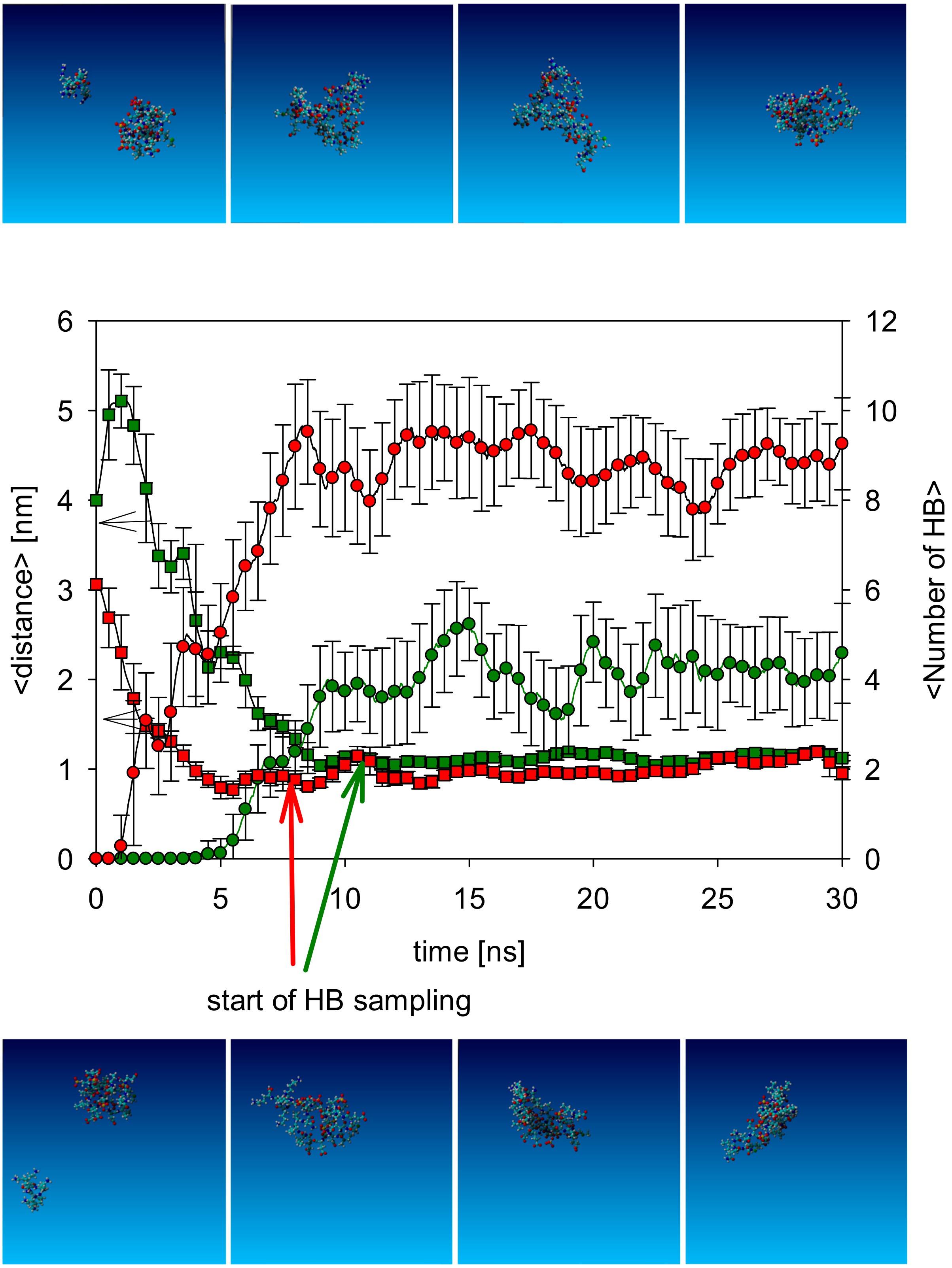

Fig. S2 The example of the evolution of the running average of Seq-A1 sequence-oligomer distance and a number of hydrogen bonds during formation of their aggregate, red symbols for arginine oligomer, green symbols – lysine oligomer. Snapshots from the simulation taken at 0 ns, 5 ns, 20 ns and 30 ns; arginine – above, lysine – below.

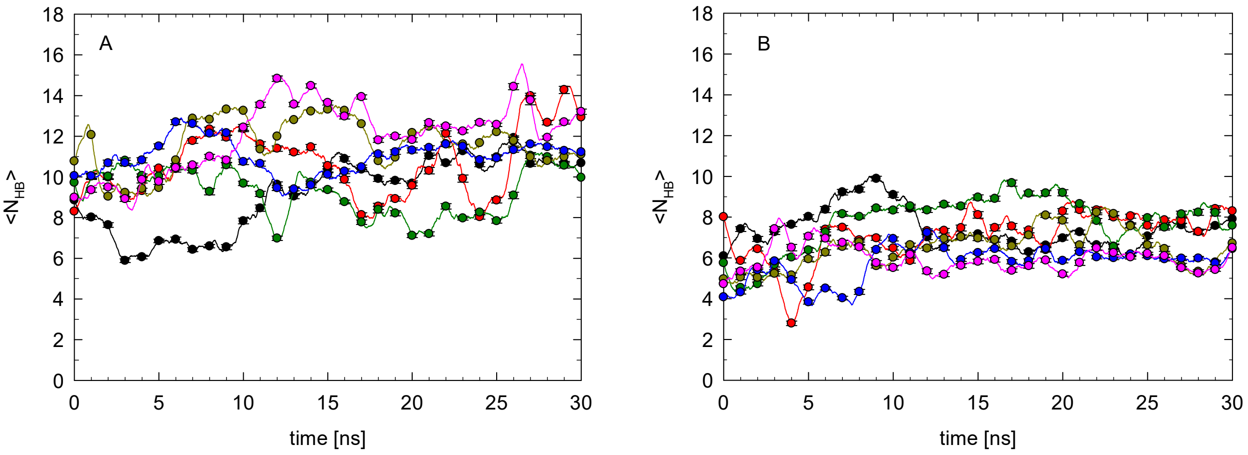

Fig. S3. The evolution of the running average of the number of hydrogen bonds in the Seq-A1 sequence-oligomer aggregates after their formation for six random initial positions. A - Seq-A1-arginine, B - Seq-A1-lysine.

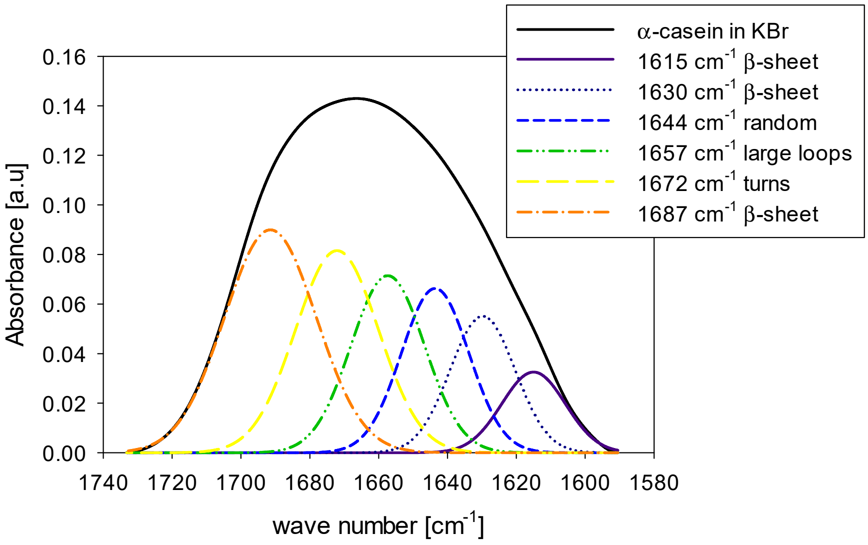

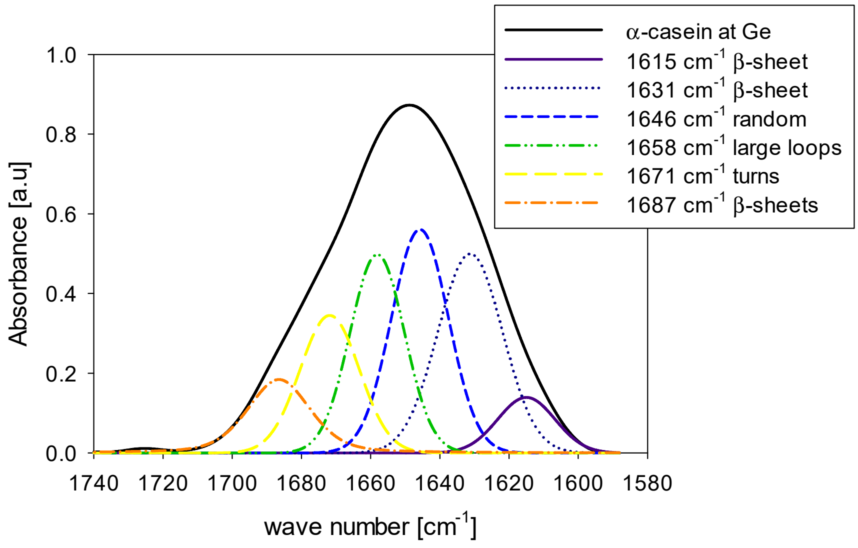

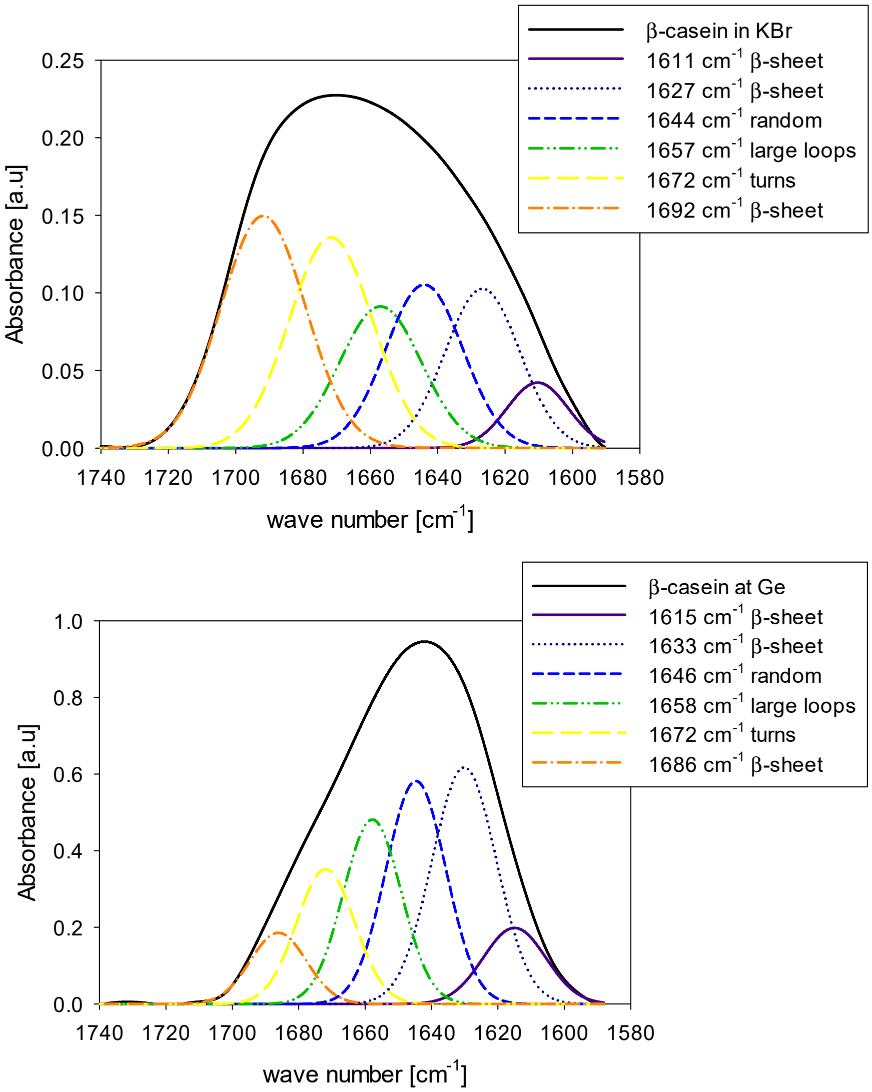

Fig. S4 Decomposition of the amide I band into structural components for α- and β-casein, measured in KBr pellets and in dried film at Ge crystal.

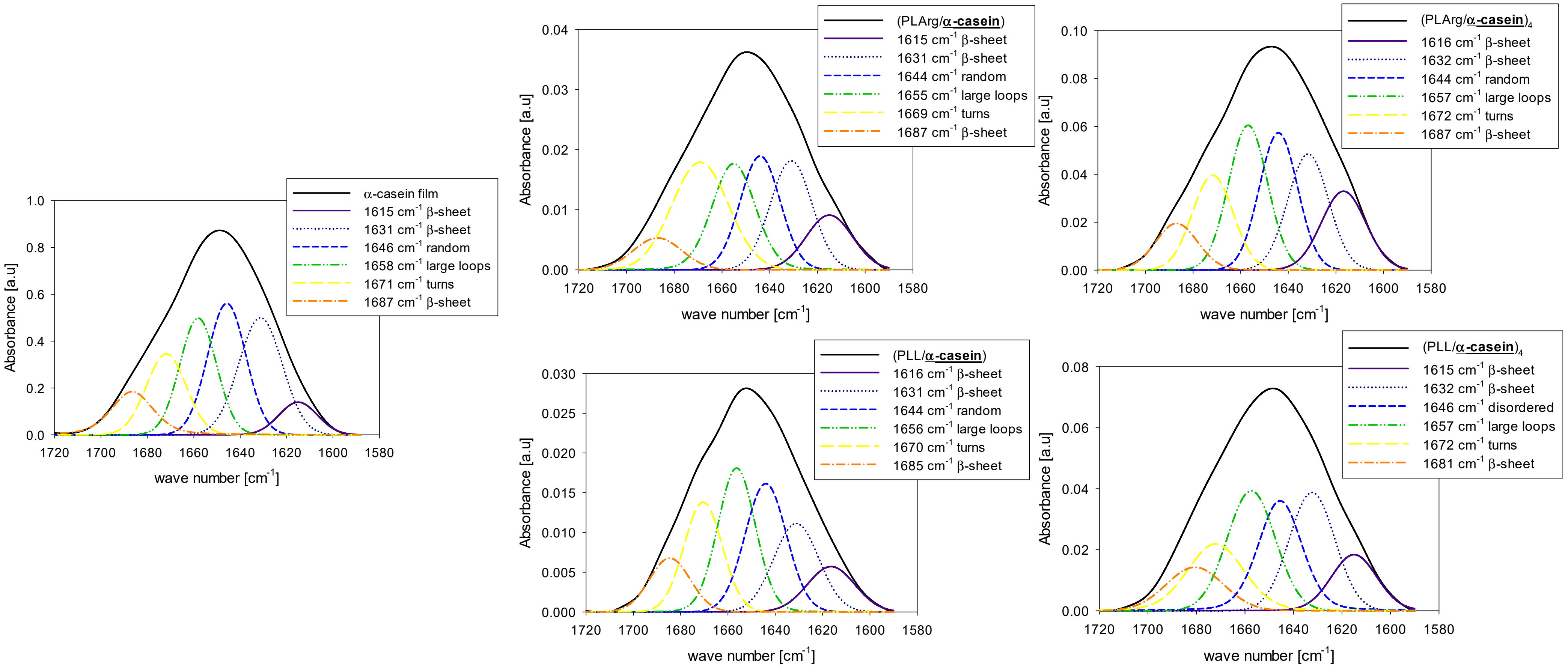

Fig. S5. Decomposition of the amide I band into structural components for α-casein film and the top α-casein layer of (PLArg/α-casein) and (PLArg/α-casein)$_4$ multilayers measured by FTIR ATR at Ge crystal.

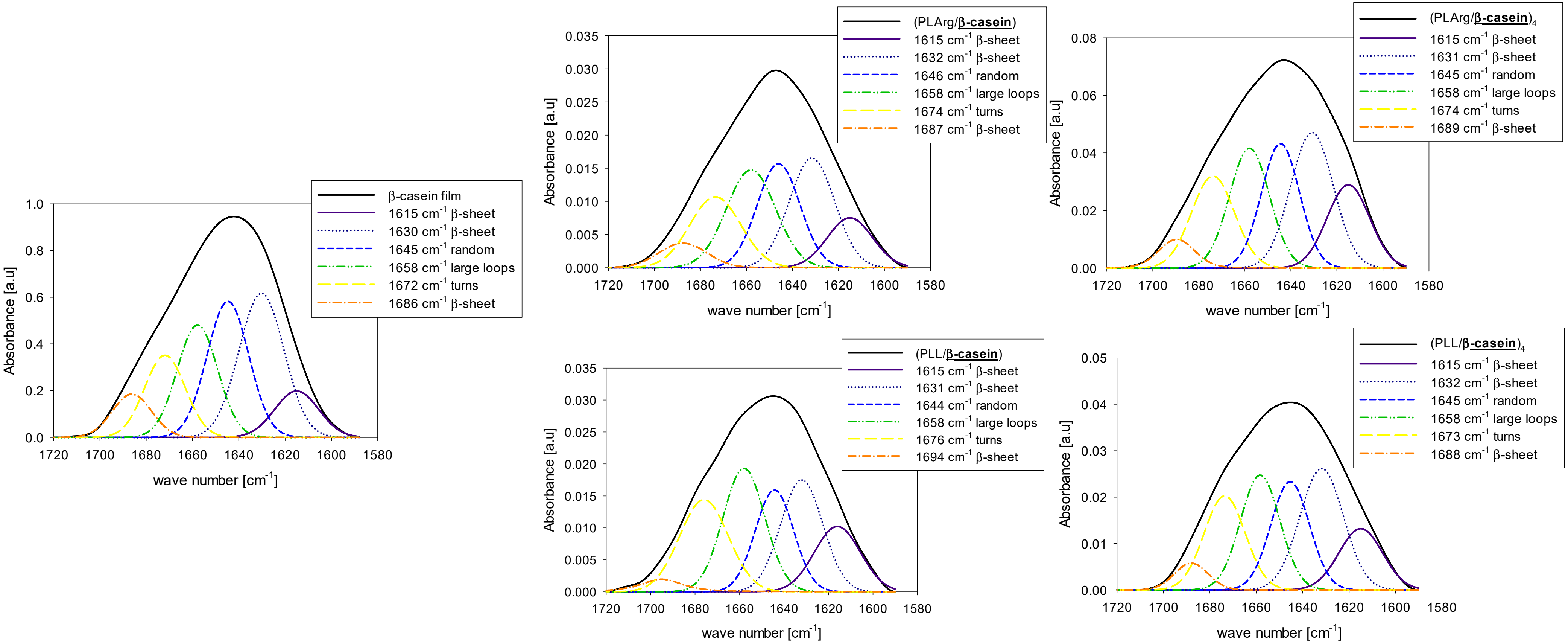

Fig. S6. Decomposition of the amide I band into structural components for β-casein film and the top β-casein layer of (PLArg/β-casein) and (PLArg/β-casein)$_4$ multilayers measured by FTIR ATR at Ge crystal
